# Nasty Viruses, Costly Plasmids, Population Dynamics, and the Conditions for Establishing and Maintaining CRISPR-Mediated Adaptive Immunity in Bacteria

**DOI:** 10.1371/journal.pgen.1001171

**Published:** 2010-10-28

**Authors:** Bruce R. Levin

**Affiliations:** Department of Biology, Emory University, Atlanta, Georgia, United States of America; University of Toronto, Canada

## Abstract

Clustered, Regularly Interspaced Short Palindromic Repeats (CRISPR) abound in the genomes of almost all archaebacteria and nearly half the eubacteria sequenced. Through a genetic interference mechanism, bacteria with CRISPR regions carrying copies of the DNA of previously encountered phage and plasmids abort the replication of phage and plasmids with these sequences. Thus it would seem that protection against infecting phage and plasmids is the selection pressure responsible for establishing and maintaining CRISPR in bacterial populations. But is it? To address this question and provide a framework and hypotheses for the experimental study of the ecology and evolution of CRISPR, I use mathematical models of the population dynamics of CRISPR-encoding bacteria with lytic phage and conjugative plasmids. The results of the numerical (computer simulation) analysis of the properties of these models with parameters in the ranges estimated for *Escherichia coli* and its phage and conjugative plasmids indicate: (1) In the presence of lytic phage there are broad conditions where bacteria with CRISPR-mediated immunity will have an advantage in competition with non-CRISPR bacteria with otherwise higher Malthusian fitness. (2) These conditions for the existence of CRISPR are narrower when there is envelope resistance to the phage. (3) While there are situations where CRISPR-mediated immunity can provide bacteria an advantage in competition with higher Malthusian fitness bacteria bearing deleterious conjugative plasmids, the conditions for this to obtain are relatively narrow and the intensity of selection favoring CRISPR weak. The parameters of these models can be independently estimated, the assumption behind their construction validated, and the hypotheses generated from the analysis of their properties tested in experimental populations of bacteria with lytic phage and conjugative plasmids. I suggest protocols for estimating these parameters and outline the design of experiments to evaluate the validity of these models and test these hypotheses.

## Author Summary

CRISPR is the acronym for the adaptive immune system that has been found in almost all archaebacteria and nearly half the eubacteria examined. Unlike the other defenses bacteria have for protection from phage and other deleterious DNAs, CRISPR has the virtues of specificity, memory, and the capacity to abort infections with a virtually indefinite diversity of deleterious DNAs. In this report, mathematical models of the population dynamics of bacteria, phage, and plasmids are used to determine the conditions under which CRISPR can become established and will be maintained in bacterial populations and the contribution of this adaptive immune system to the ecology and (co)evolution of bacteria and bacteriophage. The models predict realistic and broad conditions under which bacteria bearing CRISPR regions can invade and be maintained in populations of higher fitness bacteria confronted with bacteriophage and narrower conditions when the confrontation is with competitors carrying conjugative plasmids. The models predict that CRISPR can facilitate long-term co-evolutionary arms races between phage and bacteria and between phage- rather than resource-limited bacterial communities. The parameters of these models can be independently estimated, the assumptions behind their construction validated, and the hypotheses generated from the analysis of their properties tested with experimental populations of bacteria.

## Introduction

For many species of bacteria, adaptive evolution is through the expression of chromosomal and extrachromosomal (plasmid- and prophage - borne) genes or clusters of genes (pathogenicity and nicer islands) acquired by horizontal gene transfer (HGT) from the same or even quite distant species [1], [2]. Thus, on first consideration it may seem that bacteria and their accessory genetic elements would have mechanism to promote the acquisition, incorporation and expression of genes from without. And, indeed there are mechanisms like integrons [3]–[7] that appear to have that function. On the other side, DNA acquired from external sources may be deleterious. This is certainly the case when that DNA is borne on lytic bacteriophage, but also for plasmids that engender fitness costs [8], [9] or chromosomal DNA from the wrong source [10], [11]. To deal with these contingencies, it would seem that bacteria would have mechanisms to protect themselves against infection by deleterious foreign DNA [12]. And indeed there are systems like restriction-modification (restriction endonucleases) which appear to have that role [13], [14].

The most recently discovered mechanism postulated to provide bacteria immunity to infectious genetic elements are Clustered Regularly Interspaced Short Palindromic Repeats (CRISPR). For recent reviews see [15], [16]. CRISPR is particularly intriguing because of its ubiquity, appearing in ∼90% and ∼40% of archaeal and eubacterial sequenced genomes, respectively, and because of the adaptive mechanism by which it provides immunity to infections by a virtually indefinite diversity of bacteriophage and plasmids. DNA from infecting phage and plasmids is incorporated into the CRISPR array. Through a yet to be fully elucidated mechanism, bacteria abort the replication of infecting phage [17] or the establishment of conjugative plasmids [18] bearing copies of the DNA incorporated into their CRISPR arrays, also see [19]. Further support for CRISPR being an adaptive immune system that is maintained because it protects bacteria from infection with phage comes from studies of the community ecology of bacteria and phage; DNA in the CRISPR regions of the bacteria from those communities corresponds to that in the co-existing phage [20]–[23]. For an intriguing perspective on CRISPR as a witness to the coevolutionary history of bacteria and phage, see [24].

CRISPR-mediated immunity has been likened to a Lamarckian mechanism [25], because the selection pressure, the infecting phage and plasmids, determine the genotype. This analogy however does not account for the evolution and maintenance of the machinery responsible for taking up the infecting phage and plasmid DNA and the mechanism employed to prevent the replication or establishment of infecting genetic elements with those sequences. Under what conditions will adaptive immunity to phage and plasmid infection be the selection pressure responsible for establishing and maintaining CRISPR-mediated immunity in populations of archeae and bacteria? What about other mechanisms of resistance, like structural modification blocking phage adsorption (envelope resistance) and restriction-modification? How do these mechanisms interact with CRISPR – acquired immunity and contribute to its establishment and maintenance?

To address these questions and provide a framework and hypotheses for their study experimentally, I use mathematical models of the population dynamics of bacteria, phage and plasmids to explore the conditions under which a CRISPR–like adaptive immune mechanism will provide bacteria a selective advantage in competition with bacteria without this immune system. The results of the numerical analysis of the properties of these models suggest that with bacterial replication and phage infection parameters in realistic ranges, there are broad but not universal conditions where a CRISPR–like adaptive immune system can be favored and will be maintained in populations of bacteria confronted with lytic phage. While this model predicts conditions where CRISPR-mediated immunity will be favored when bacteria compete with populations bearing conjugative plasmids, these conditions are relatively restrictive. The parameters of these models can be independently estimated, the validity of the assumptions behind their construction and the hypotheses generated from the analysis of the properties can be tested in experimental populations of bacteria with lytic phage and conjugative plasmids. Procedures for doing these experiments are outlined and their potential outcomes described and/or speculated upon. Also discussed are the broader implications of CRISR-mediated adaptive immunity to the population and evolutionary biology and ecology of bacteria and phage.

## Model

### Bacterial growth and population maintenance

Both the lytic phage and conjugative plasmid models used here assume a chemostat-like habitat. The bacteria grow at a rate that is a monotonically increasing function of the concentration of a limiting resource, R µg/ml [26].
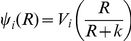
where V_i_ hr^−1^ is the maximum growth rate of the i^th^ strain of bacteria and *k* the concentration of the resource when the growth rate is half its maximum value (the “Monod constant”). The populations are maintained in a vessel of unit volume, (1ml) into which medium containing the limiting resource from a reservoir where it is maintained at a concentration *A* µg/ml flows in at a rate *w* per hour. Excess resource and wastes are removed from the vessel at the same rate. As in [27], the rate of uptake of the resource by the bacteria is proportional to the density, the resource concentration-dependent growth rates of the different populations of bacteria and a conversion efficiency parameter, *e* µg/per cell.

### The phage model

The model developed here is an extension of that in [28]. There are four populations of bacteria. Two are sensitive to the phage, *N*, non–CRISPR and *C*, CRISPR and two that are either fully resistant (envelope resistance), or immune because of CRISPR, *N_R_* and *C_R_*, respectively. The variables *N*, *C*, *N_R_* and *C_R_* are the both the densities (bacteria per ml) of these populations and used as their designations. There is one population of phage, with density and designation, *P* particles per ml.

**Figure 1:**
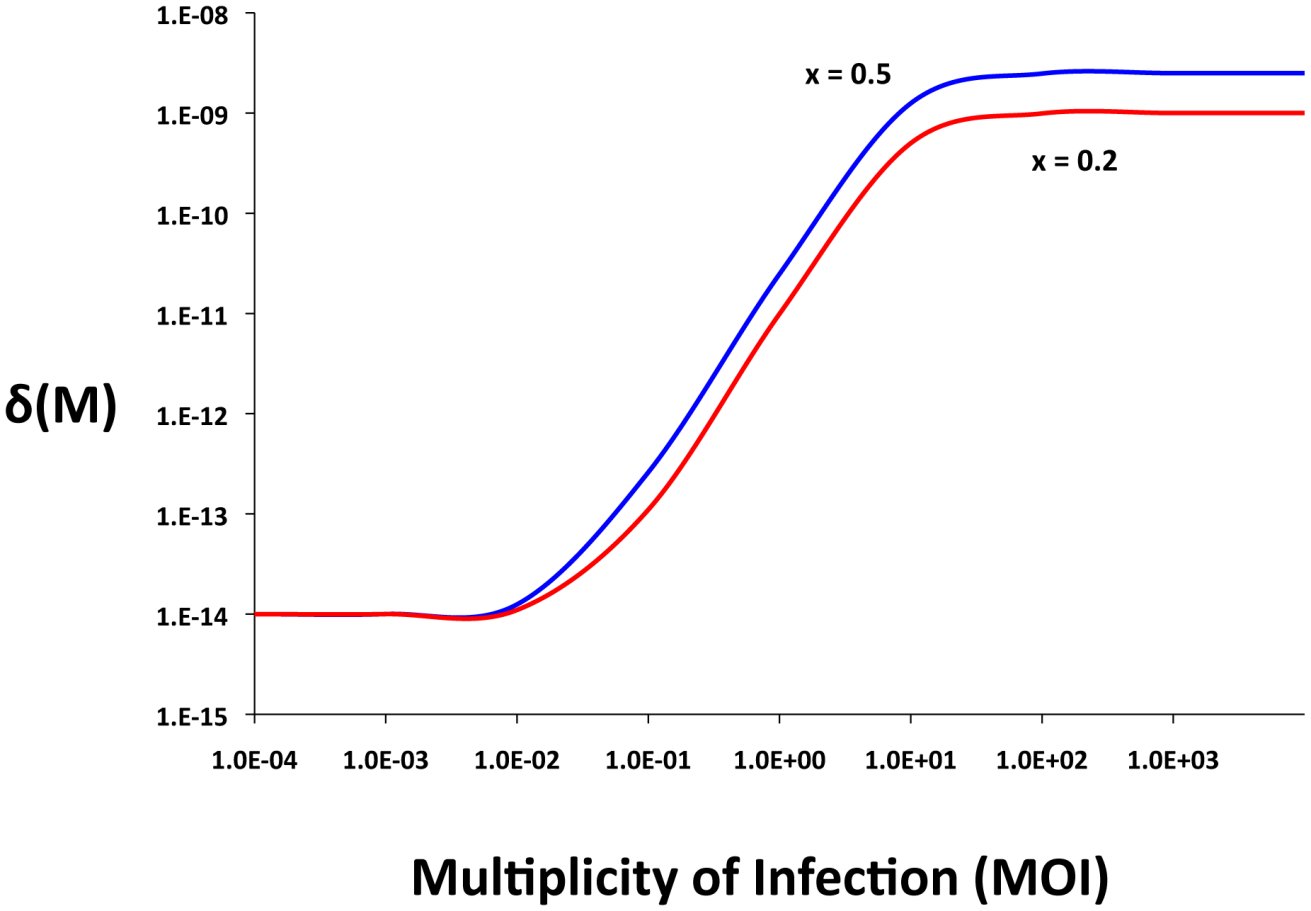
Adsorption rate as a function of the multiplicity of infection (MOI), δ_MIN_ = 10^−14^, δ_MAX_ = 5×10^−9^, *x* = 0.5, or x = 0.2 *q* = 10^2^, and *n* = 2.

The phage adsorb to the *N* and *C* and *C_R_* bacteria with rate constants, *δ_N_ and δ_C_* (ml per phage per cell per hour) respectively. Phage do not adsorb to bacteria with envelope resistant, i.e. the *N_R_* cells. To account for a possible multiplicity of infection (MOI) effect on survival of phage-infected C_R_, the effective killing rate constant for phage adsorption to CRISPR can be an increasing function of the ratio of free phage and *C_R_* cells, *M = P/C_R_*.
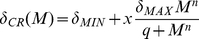
(1)where δ_MIN_ and δ_MAX_ are the minimum and maximum adsorption rates. The parameter *x* is a coefficient (0≤*x*≤1) that specifies the magnitude of the MOI effect, *q* is the MOI where the adsorption rate is half its maximum value and *n* is an exponent which contributes to the shape of the distribution. At low multiplicities, *δ_CR_ (M)* the CRISPR cells would be effectively immune (resistant) (Figure 1). At high multiplicities, however, immune CRISPR cells can be overburdened by phage, their immunity would be overridden, and the phage would replicate, killing the cells. On the other side, we assume that the phage are removed from the population by adsorption to immune CRISPR cells at the maximum adsorption rate, δ_MAX_.

For convenience I neglect the latent periods of the phage infection but assume that the phage have potentially different burst sizes, *β_N_*, *β*_C_, and *β_CR_* particles per cell, for *N*, *C* and *C_R_* cells, respectively.

Phage-immune CRISPR cells, *C_R_* are produced from *C* at a rate proportional to the rate at which the phage adsorb to them and a constant *m* (0≤*m*≤1) which is the probability that a phage infection will be aborted and a CRISPR strain will be produced. At a rate *v* per cell per hour, CRISPR lose their immunity, *C_R_→C*. For the *N* and *C* populations the loss of the adsorbed phage is subsumed in the value of the burst size (which is one less than the number of phage produced). For the *C_R_* population, the loss of the phage due to adsorption is specifically considered because only a small fraction of the adsorbed phage replicate when the MOI is low.

**Figure 2:**
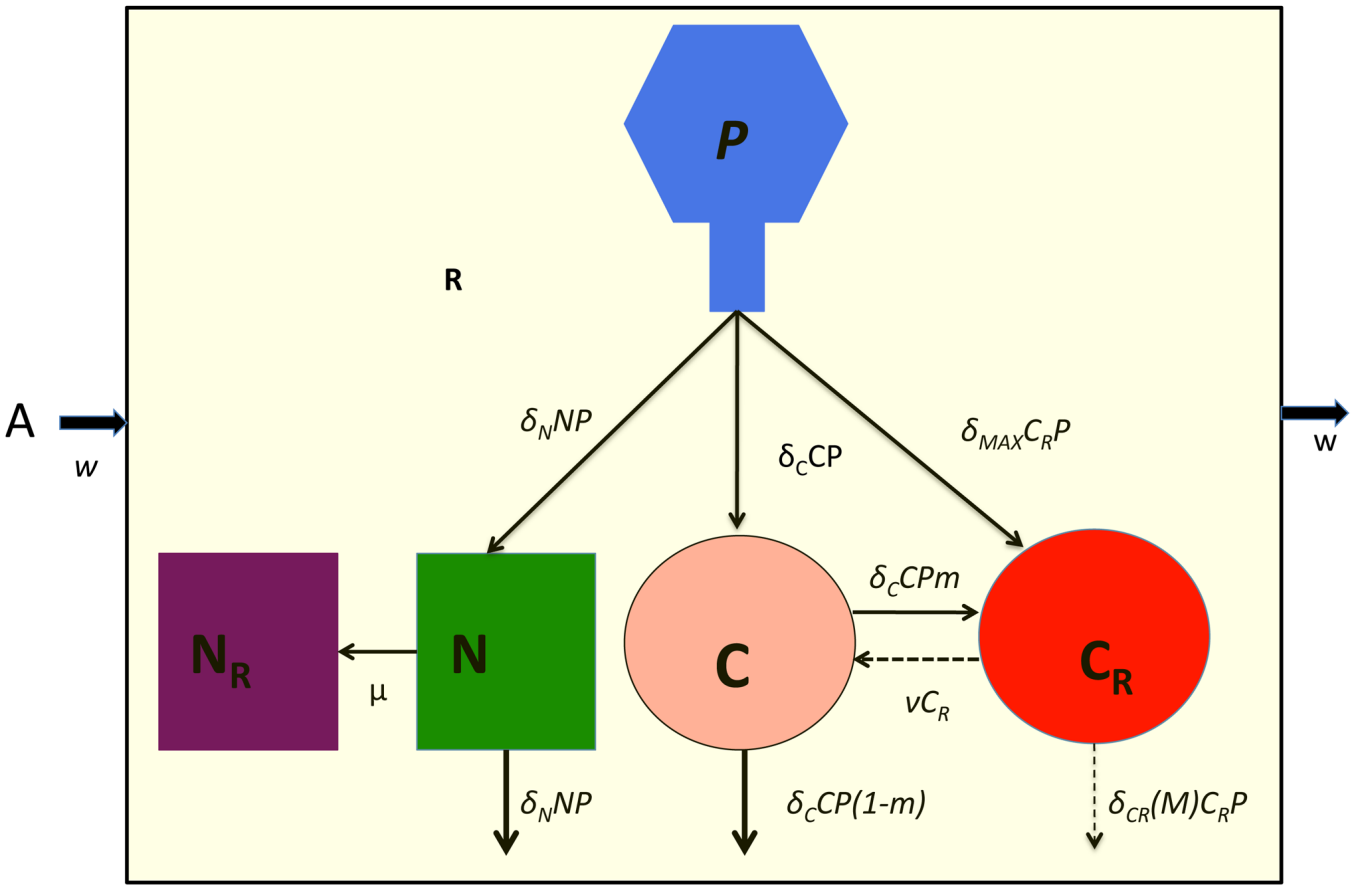
Model of the population dynamics of lytic phage with CRISPR-mediated adaptive immunity and envelope resistance in continuous culture: *P* – phage, *N* – phage sensitive non–CRISPR bacteria, *N_R_* – envelope resistant, non–CRISPR bacteria C - phage sensitive CRISPR bacteria, *C_R_* - phage immune CRISPR bacteria. The δs are the adsorption rate constants, *m* is the fraction of C to which phage are adsorbed that enter the immune state, ν is the rate at which immune CRISPR cells lose their immunity, and μ is the rate of mutation to envelope resistance. While the phage adsorb to immune CRISPR cells at the maximum rate and are removed from the phage population, their replication on CRISPR cells and the rate of mortality of immune CRISPR is either 0 or a monotonically increasing function of the multiplicity of infection (equation (1)). The bacteria reproduce at a rate proportional to the concentration of a limiting resource and their maximum rates of replication. Phage replication is through the killing of adsorbed bacteria and their burst size, β, on that cell line. The limiting resource in the reservoir is at concentration A µg/ml and enters the vessel at a rate, *w*, which is the same rate at which the phage and bacterial populations and excess resource, *R*, are removed from the vessel. For more details see the text.

In Table 1, I separately define these parameters and in Figure 2, illustrate the interactions between the different populations of bacteria and the phage. The equations for this model follow.
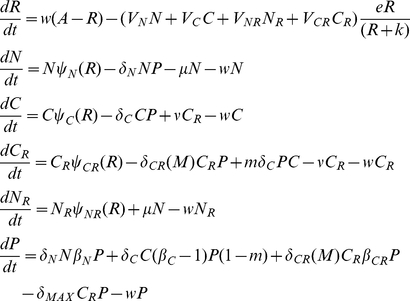



**Table 1:** Phage model variables and parameters.

Variable* or Parameter+	Definition	Parameter	Definition
*N*	P Sensitive N-C	*w*	Dilution rate
*N_R_*	P Resistant N-C	*δ_N_*	Adsorption rate P to N
*C*	P Sensitive C	*δ_C_*	Adsorption rate P to C
*C_R_*	P Immune C	*δ_MIN_*	Min. Adsorp. Rate P to C_R_
*P*	Phage	*δ_MAX_*	Max. Adsorp. rate P to C_R_
*R*	Resource Conc.	*x*	Multiplicity Coef. P to C_R_
*V_N_*	Max. Growth N	*n*	Multiplicity Exp. P to C_R_
*V_NR_*	Max. Growth N_R_	*q*	Multiplicity half Max Density
*V_C_*	Max. Growth C	*β_N_*	Bursts size P on N
*VC_R_*	Max. Growth C_R_	*β_C_*	Burst size P on C
*K*	Monod Constant	*β_CR_*	Burst size P on C_R_
*E*	Conversion Effic.	*μ*	Mutation rate N to N_R_
*A*	Reservoir Conc. R	*m*	Fraction of infected C→C_R_
		*v*	Rate of Loss of immunity C_R_→C

N-C – Non CRISPR, C – CRISPR.

*The variables are densities of bacteria or phage per ml or the concentration of the resource, µg per ml.

+See the text for the dimensions of the parameters.

### The conjugative plasmid model

**Figure 3:**
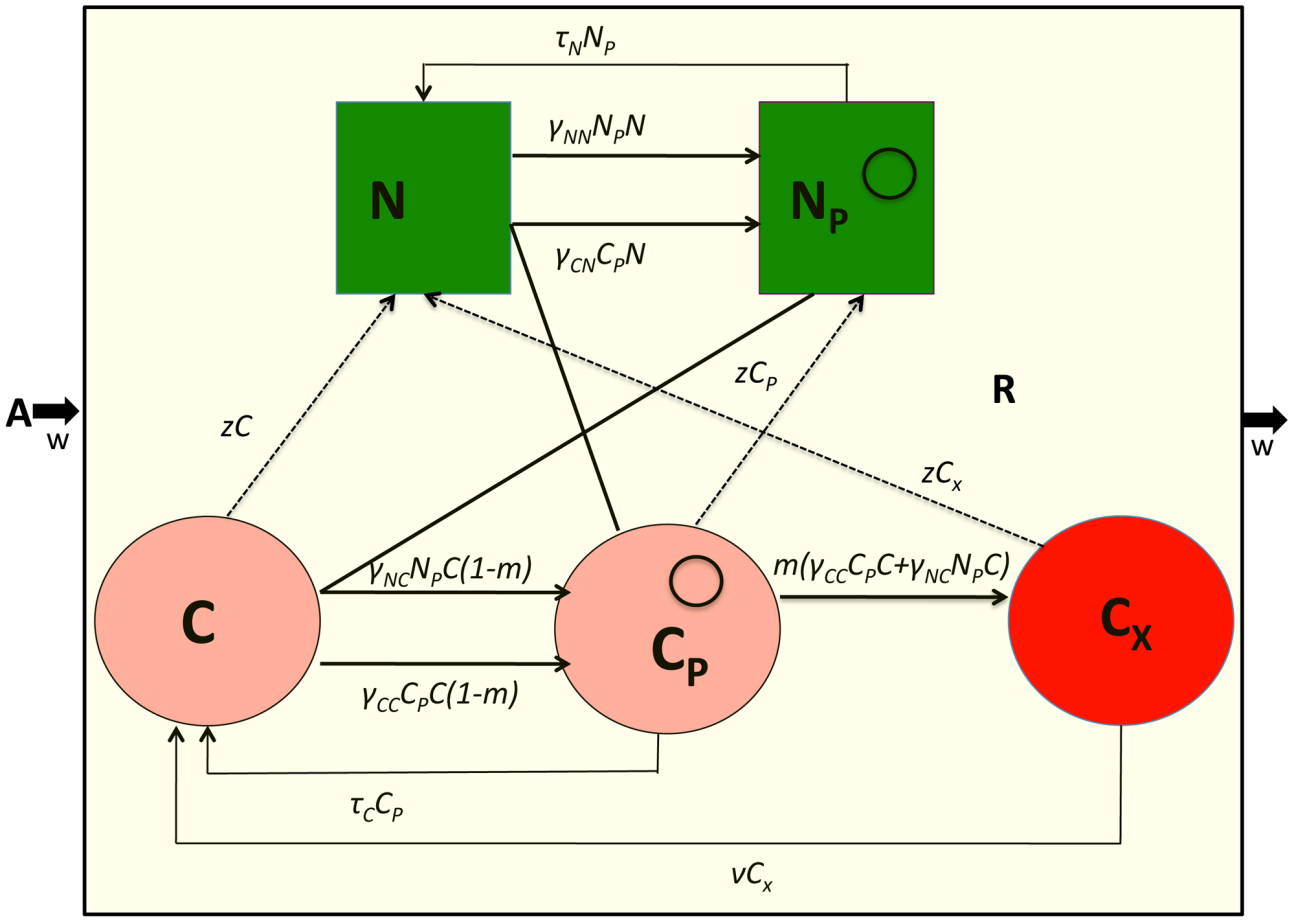
Model of the population dynamics of a conjugative plasmid with CRISPR-mediated adaptive immunity in continuous culture. *N* - plasmid-free non–CRISPR, *N_P_* - plasmid-bearing non–CRISPR, *C* - plasmid-free CRISPR, *C*_P_ - plasmid-bearing CRISPR, C*_X_* - immune CRISPR. The γs are the rate constants of plasmid transfer, *m* is the fraction of *C_P_* that enter the immune state C_X_ upon receiving the plasmid from an N_P_ or C_P_, ν is the rate at which immune CRISPR cells lose their immunity and *z* the rate at which the CRISPR cells lose the CRISPR element and become N or N_P_. The bacteria reproduce at a rate proportional to the concentration of a limiting resource and their maximum rates of replication. The limiting resource in the reservoir is at concentration A µg/ml and enters the vessel at the rate, w, which is the same as the rate at which the phage and bacterial populations and excess resource, R, are removed from the vessel. For more details see the text.

The model developed here is an extension of that in [29]. There are five bacterial populations. Two populations do not code for CRISPR, *N* and *N_P_*, and three populations code for CRISPR, *C* and *C_P_* and *C_X_*. The *N_P_* and C_P_ populations bear the conjugative plasmid and *C_X_*, carries CRISPR and plasmid sequences that make it completely immune to the receipt of these plasmids. Plasmids are transferred by conjugation at rates proportional to the product of the densities of the plasmid-bearing and plasmid-free populations and rate constants, *γ_NN_*, *γ_NC_*, *γ_CN_* and *γ_CC_* (ml per cell per hour) respectively for the transfer of the plasmid from *N_P_* to *N*, *N_P_* to *C*, *C_P_* to *N* and *C_P_* to *C*., respectively. Plasmids are lost by vegetative segregation at rates *τ_N_* and *τ_C_* per cell per hour, with *N_P_→N* and *C_p_→C*. *C* are converted to C_X_ at a rate proportional to the rate at which C acquires the plasmid and a probability *m* (0≤*m*≤1). *C_x_* lose the CRISPR plasmid immunity region and become C at rate ν per cell per hour. Each of the cell lines, have a maximum growth rate, *V_N_*, *V_NP_*, *V_C_*, and *V_CP_*, and *V_X_* per hour. In Figure 3, I illustrate the interactions between the different cell lines in this model, and, in Table 2, I separately define the parameters and variables. The equations for this model are:
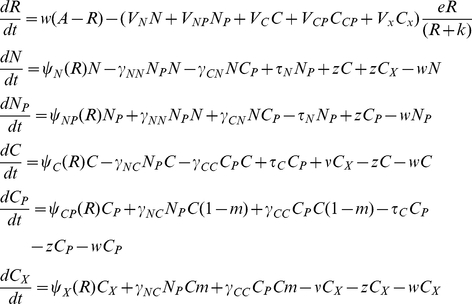



**Table 2:** Plasmid model variables and parameters.

Variable* or Parameter+	Definition	Parameter	Definition
*N*	Plasmid-free N-C	*e*	Conversion efficiency
*N_P_*	Plasmid-bearing N-C	*γ_NN_*	Pl rate constant N_P_ to N
*C*	Plasmid-free C	*γ_NC_*	Pl rate constant N_P_ to C
*C_P_*	Plasmid-bearing C	*γ_CN_*	Pl rate constant C_P_ to N
*C_X_*	Immune C	*γ_CC_*	Pl rate constant C_P_ to C
*R*	Resource Conc.	*τ_N_*,	Pl Segreg. Rate N_P_ to N
*V_N_*	Max. Growth N	*τ_C_*	Pl Segreg. Rate C_P_ to C
*V_NP_*	Max. Growth NP	*υ*	Rate of loss of Immunity C_X_ to C
*V_C_*	Max. Growth C	*m*	Fraction of infected C become C_X_
*V_CX_*	Max. Growth CP	*w*	Dilution rate
*V_X_*	Max. Growth X	*A*	Resource Conc. Reservoir
*k*	Monod Constant	*z*	Rate of loss of CRISPR into N or N_P_

N-C Non–CRISPR, C- CRISPR.

*Variables are bacteria per ml or for the resource µg/ml.

**+:** See the text for the dimensions of the parameters.

### Numerical solutions

For the numerical solutions to these equations (computer simulations) I use a differential equation-solving software package, Berkeley Madonna. For the phage simulations there is a refuge density, below which the phage are unable to adsorb to the bacteria. The purpose of this is to control the system from oscillating without limits, see [30]. In these simulations, if the phage density falls below 10^−1^ particles per ml, the phage are considered to be lost. Copies of these simulations are available online, www.eclf.net/programs.

## Results

### The population dynamics and evolution of CRISPR bacteria with phage

The bacterial growth, resource-uptake, phage adsorption parameters and burst sizes used in these simulations (Table 1) are in a range similar to that which we observed for *E. coli* and the phages T2 and T7 [28], [31].

#### Invasion and maintenance of CRISPR in the absence of envelope resistance

**Figure 4:**
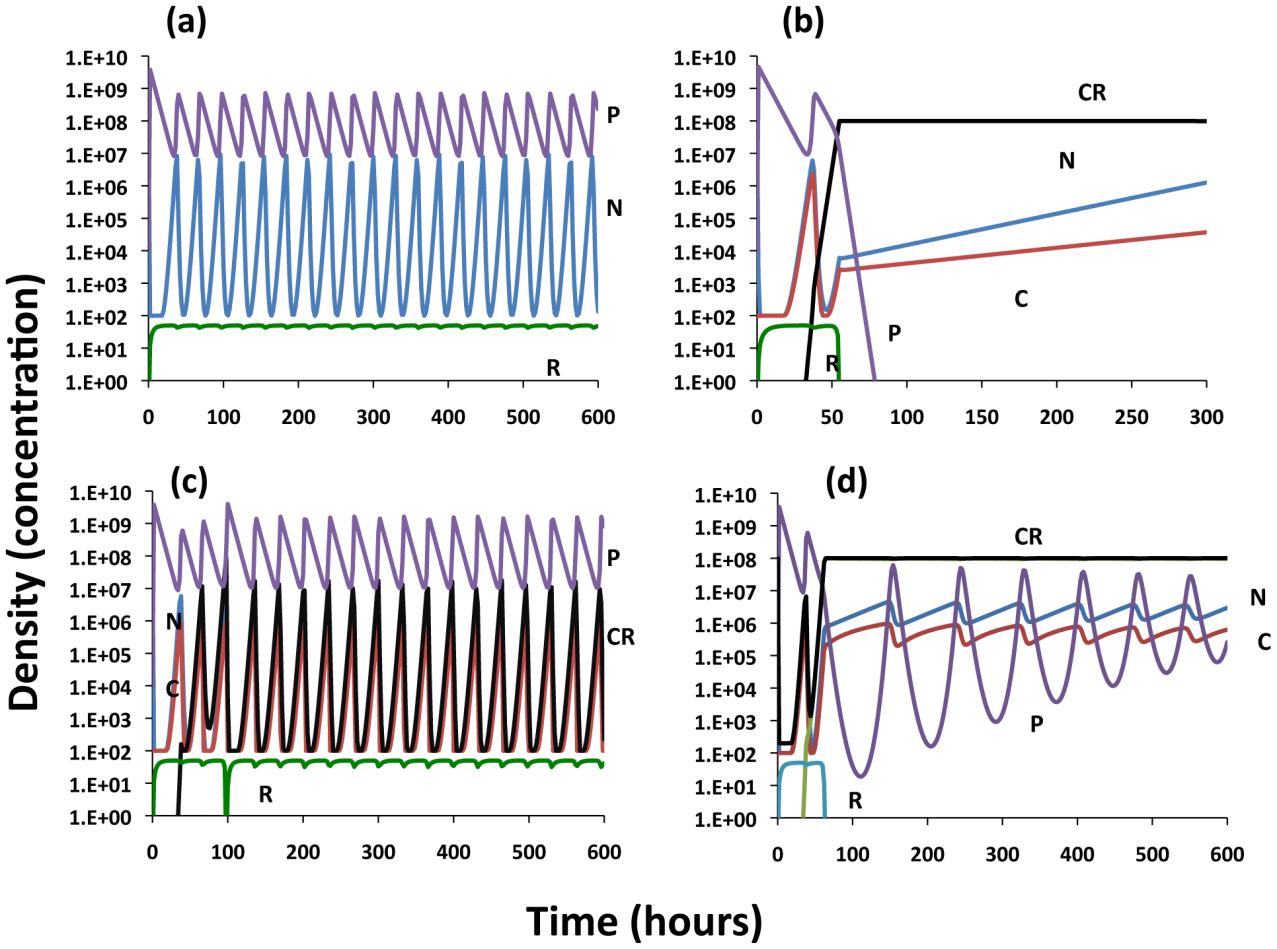
Population dynamics of lytic phage, *P*, with sensitive non–CRISPR bacteria, *N*, non-immune and immune CRISPR-encoding cells, C and C_R_, respectively. Changes in the densities of the bacterial and phage populations and the concentration of the limiting resource, R. In this and the other simulations, A = 50 µg/m, w = 0.2 per hour, e = 5×10^−7^µg, k = 0.25 µg. In these phage simulations, β_N_ = β_C_ = **β_CP_**. (a) The dynamics of sensitive bacteria and phage in the absence of CRISPR, V_N_ = 1.0 hr^−1^, δ_N_ = 5×10^−9^. (b) Invasion of CRISPR in the presence of phage, no MOI effect (x = 0), V_N_ = 1.0. V_C_ = 0.95, V_CR_ = 0.90, *δ_N_* = *δ_C_* = 5×10^−9^, *δ_CP_* = *δ_MIN_* = *10^−14^* (*δ_MAX_ = 5×10^−9^*) (c) Invasion of CRISPR with presence of phage V_N_ = 1.0. V_C_ = 0.95, V_CR_ = 0.90, *δ_N_* = *δ_C_* = 5×10^−9^, Strong MOI effect (x = 0.5, n = 2.0, q = 10^2^, *δ_MIN_* = 10^−14^, *δ_MAX_* = 5×10^−9^). (d) Invasion of CRISPR with presence of phage, V_N_ = 1.0. V_C_ = 0.95, V_CR_ = 0.90, *δ_N_* = *δ_C_* = 5×10^−9^, Modest MOI effect (x = 0.2, n = 2.0, q = 10^2^, *δ_MIN_* = 10^−14^, *δ_MAX_* = 5×10^−9^).

In a chemostat with susceptible bacteria at an equilibrium density N*, a lytic phage can become established and will maintain a population with sensitive bacteria as long as the rate of phage production exceeds the rate of washout, δ_N_β_N_N*>w [28]. With the parameters used in these simulations, N^*^∼10^8^ (see [32]). As long as δ_N_β_N_N^*^>2×10^−9^, the phage will become established and can maintain a population by replicating on sensitive bacteria (Figure 4A). The oscillations in the densities of bacteria and phage in these and the following simulations are those anticipated for the predator-prey nature of these dynamics.

To explore the conditions under which a CRISPR population will become established and be maintained in the presence of phage, I consider situations where the *C* and *C_R_* populations have an intrinsic selective disadvantage relative to *N* (*V_N_>V_C_*, *V_CR_*) and therefore cannot invade an established *N* population in the absence of these bacterial viruses. Because of the immunity of *C_R_*, with phage present and in the absence of a multiplicity effect, an initially rare CRISPR population will invade and ascend to dominance despite its lower intrinsic fitness (Figure 4B). With these parameters, the phage are maintained along with *N* and *C*, the latter being continually generated by the loss of immunity by the dominant C_R_ population. The *N* population is maintained because of its higher intrinsic fitness (growth rate) relative to *C_R_*, and resources, rather than phage predation, limit the bacteria at large. The phage continue to be maintained by replicating on the N and C cells. Although the oscillations are damped and in time would no longer be noticed, that time would be considerably greater than would be feasible to study experimentally with chemostats. If we allow for a strong multiplicity effect (x = 0.5), the CRISPR population becomes established, and both immune and non-immune CRISPR cells maintain their populations with sensitive non–CRISPR in a phage- rather than resource- limited community (Figure 4C). When the magnitude of the multiplicity effect is reduced (x = 0.2), the phage continue to be maintained but immune CRISPR cells ascend to dominance and the community with three populations of bacteria, N, C and C_R_ are maintained in a resource- rather than a phage-limited state (Figure 4D).

#### The invasion and maintenance of CRISPR in the presence of envelope resistance

In addition to CRISPR immunity, when confronted with phage, bacteria may generate mutants to which phage are unable to adsorb or are resistant by other mechanisms [33]. To explore how this envelope resistance will affect the conditions for the establishment and maintenance of CRISPR, we consider the invasion of an envelope resistant strain of *N*, *N_R_*, into a population of *N* and phage. In these simulations, the C and C_R_ are less fit than *N* (*V_N_>V_C_*, *V_CR_*) and *N_R_* are less fit than *C* and *C_R_*, (*V_NR_<V_C_*, *V_CR_*). Were the *N_R_* cells more fit than *C* and *C_R_*, they would dominate and the CRISPR population would not invade an would not be established. Whether this fitness relationship will be seen with real bacteria and what those fitness will be is an empirical question.

**Figure 5:**
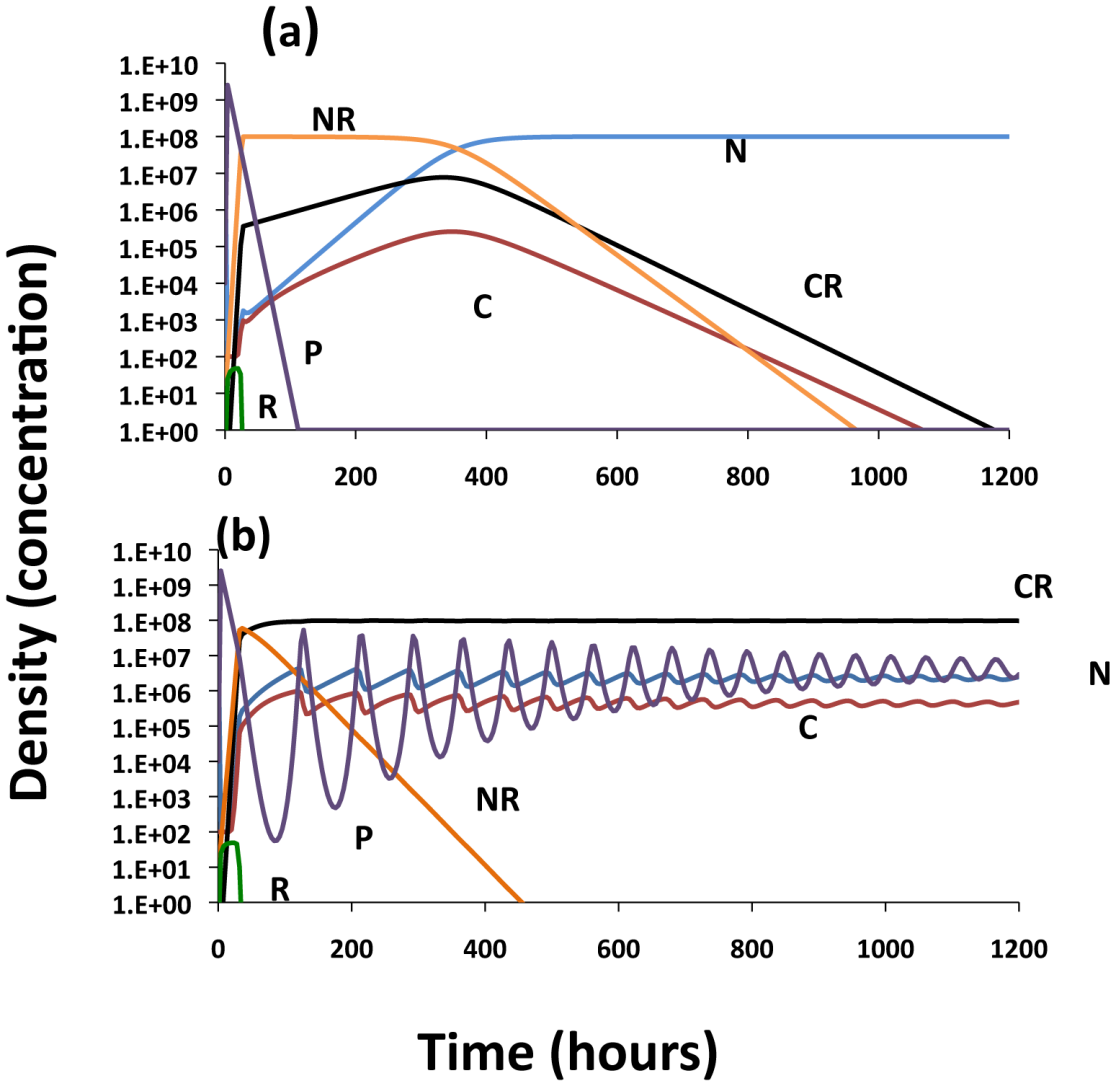
Population dynamics of lytic phage, *P*, with sensitive and resistant non–CRISPR bacteria, *N* and N_R_, non-immune and immune CRISPR-encoding cells, C and C_R_, respectively. Changes in the densities of the bacterial and phage populations and the concentration of the limiting resource, R. Unless otherwise noted, the parameter values used are those in Figure 4B. (a) Invasion of C and N_R_ into a population with phage, modest cost of resistance, V_NR_ = 0.85. (b) Invasion of C and N_R_ into a population with phage, with a greater cost of resistance, V_NR_ = 0.70.

As can be seen in Figure 5A, although the resistant, *N_R_* strain is the least intrinsically fit bacteria in the community (lowest maximum growth rate), in the presence of phage it ascends rapidly and achieves dominance. During this initial phase, as a consequence of the production of immune *C_R_* cells, the CRISPR population also increases in density, but remains a minority population relative to the resistant non–CRISPR N_R_. With these parameters, the phage density declines after the ascent of resistance and the densities of both the *N* and *C* populations increase. Shortly after the phage are eliminated the highest fitness *N* population ascends and lower fitness *C*, *C_R_* and *N_R_* decline. If the phage resistant population is substantially less fit than the other bacterial populations, the C_R_ population ascends to dominance and continues to co-exist with the phage, *N*, and *C* populations (Figure 5B).

### The population dynamics of CRISPR with conjugative plasmids

In accord with [34], conjugative plasmids will be maintained as long as the rate of infectious transfer exceeds the rates of loss of the plasmid due to selection against the cells carrying it, vegetative segregation, and the rate of flow through the chemostat. In terms of the above parameters, the plasmid will be maintained in an *N-NP* population as long as
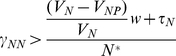
(2)where N^*^ is the density of plasmid-free cells at the chemostat equilibrium. For example, if V_N_ = 1.0, V_NP_ = 0.95, w = 0.2, τ_N_ = 10^−3^, the plasmid will be maintained in a population of density N^*^ = 10^8^ as long as *γ_NN_*>1.1×10^−10^. If the plasmid augments the growth rate (which in this model is the sole parameter of cell fitness) of the bacteria that carry it, *V_NP_*>*V_N_*, as we would anticipate for antibiotic resistance encoding plasmids in the presence of the selecting antibiotic, bacteria bearing the plasmid will be able to invade even without transfer, as long as the segregation rate, τ_N_, is sufficiently small.

#### Invasion and maintenance of CRISPR in the presence of a competing population bearing a conjugative plasmid

**Figure 6:**
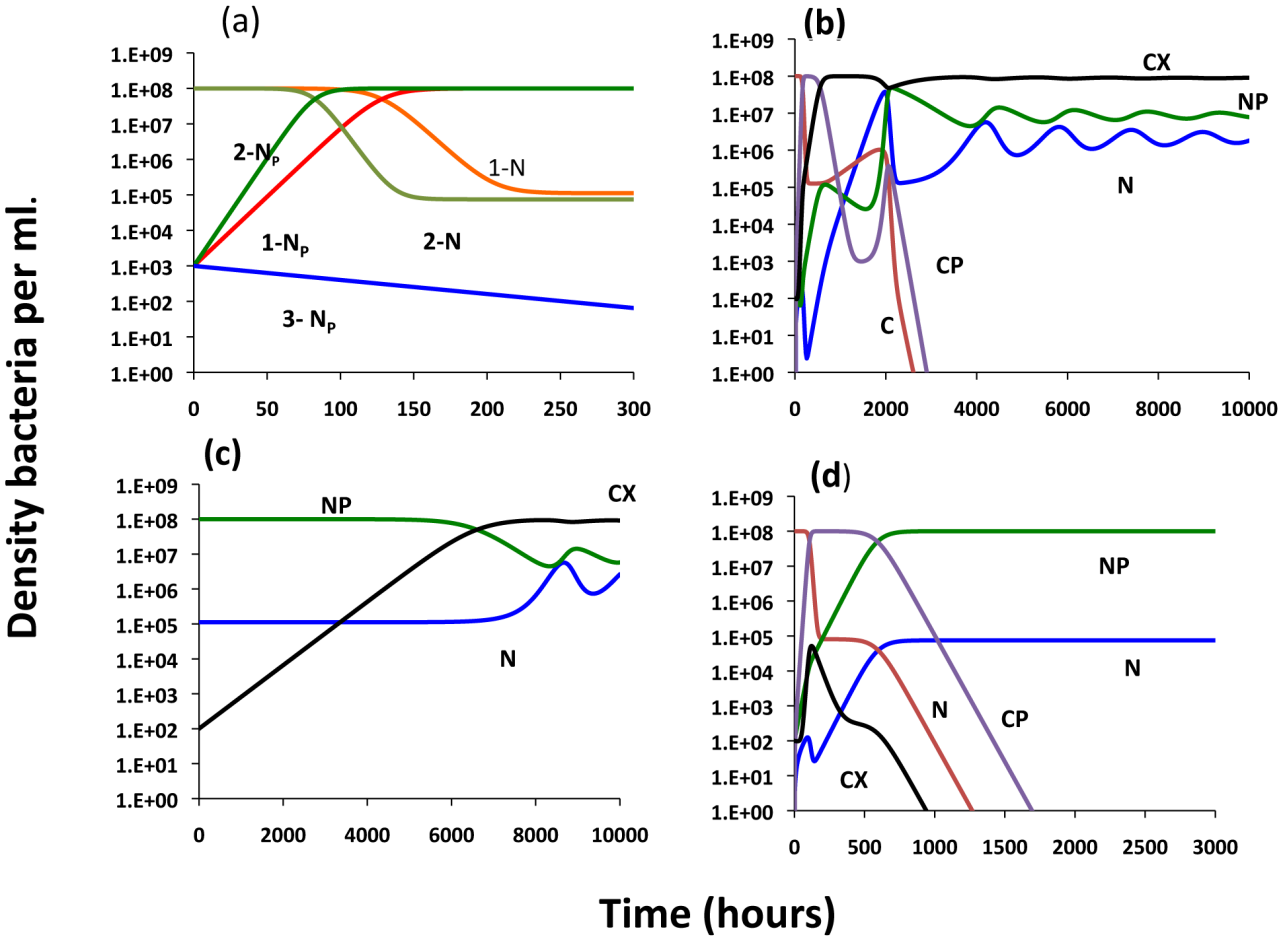
Population dynamics of a conjugative plasmid with non–CRISPR, N and N_P_ and CRISPR, C, C_P_ and C_X_ populations; changes in the densities of the bacterial populations. Unless otherwise noted all of the rate constants of plasmid transfer, the γ_ij_s = 10^−9^ [38], the segregation rates, τ_N_ and τ_C_ = 10^−3^, the rate of loss of immunity ν = 10^−3^, upon receiving the plasmid the rate of conversion of C_P_ to C_X_ = 0.2, and the rate of conversion of CRISPR cells to N or N_P_, z = 10^−8^. (a) No CRISPR – Just N and N_P_ 1 - Deleterious plasmid V_N_ = 1, V_NP_ = 0.95; 2 - a beneficial plasmid V_N_ = 1, V_NP_ = 1.2 and 3- deleterious plasmid V_N_ = 1, V_NP_ = 0.95, γ_NN_ = 10^−11^. (b) Invasion of bacteria carrying a deleterious plasmid into a lower fitness CRISPR, C, population, V_N_ = 1, V_NP_ = 0.95, V_C_ = 0.97, V_CP_ = 0.88, V_x_ = 0.96, (c) Invasion of CRISPR X into a equilibrium population of plasmid-bearing and plasmid free cells, N-NP with a deleterious plasmid (parameters the same as b). (d) Invasion of cells carrying a higher fitness plasmid, NP, into a C population, V_N_ = 1, V_NP_ = 1.2 V_C_ = 0.97, V_CP_ = 1.1, V_x_ = 0.96.

The population dynamics of selection and plasmid transfer in an equilibrium chemostat in the absence of CRISPR are presented in Figure 6A. If the conditions specified in equation (2) are met, the plasmid- bearing cells become established and ascend to dominate the *N-NP* community, whether cells bearing the plasmid are favored or not. If the plasmid is maintained by transfer or selection for the genes it carries and τ_N_>0, there will be a stable population of plasmid-free cells. When the rate constant of plasmid transfer is too low, the deleterious plasmid will be lost.

To consider the effects of CRISPR on the population dynamics of bacteria with conjugative plasmids and the conditions under which CRISPR immunity will provide an advantage to bacteria, I let the maximum growth rates of the CRISPR strains (the sole measure of intrinsic, phage-independent fitness) be somewhat lower than the corresponding non–CRISPR cells. In Figure 6B, the population is initially at equilibrium with a plasmid-free, non-immune CRISPR population and a low density of plasmid-bearing non–CRISPR bacteria are introduced. The plasmid spreads rapidly from N_P_ to C producing a *C_P_* population which in turn generates immune CRISPR, *C_X_*. While the *C* and *C*_P_ populations die out, *C_X_* ascends to dominance and minority populations of N and N_P_ are maintained. Although the C_X_ population has a lower growth rate than N, in the presence of a deleterious conjugative plasmid they have an advantage because they cannot be infected by that element. They do not eliminate the *N* and *N_P_* populations due to the loss of the CRISPR region and the conversion into *N*. As can be seen in Figure 6C, with these parameters and a lower growth rate, *C_X_* can invade an equilibrium *N-N_P_* population, but the rate of increase in the density of C_x_ is low. The invasion rate for C_X_ would even be further reduced if, instead of C_X_, a plasmid-free C invaded an NP population, because it would be some time before the C_X_ is produced and, in a finite population, may not be produced at all (“data” not shown). A very different situation obtains when the plasmid confers a growth rate advantage to the infected host (Figure 6D). Under these conditions, the C populations and its derivatives, C_P_ and C_X_, are eliminated.

## Discussion

“All models are wrong, some are useful.” (George Box)

It has been less than eight years since the ubiquitous clusters of palindromic repeats now known as CRISPR first acquired this moniker [35]. Although there had been compelling circumstantial evidence that CRISPR was part of an adaptive immune system that provides protection against infecting phage and plasmids, it has been less than four and three years respectively since the publication of the first direct (read experimental) evidence that CRISPR can provide immunity to infection by lytic phage [17] and conjugative plasmids [18].

In the course of this time a great deal has been learned about the molecular biology of CRISPR and the mechanisms by which it provides adaptive immunity to plasmid and phage infection. But there remain many unanswered questions about these processes. Most important for this consideration is a dearth of the quantitative information needed to understand the population dynamics of CRISPR-mediated adaptive immunity and thereby the conditions for the establishment and maintenance of CRISPR in bacterial populations. To my knowledge, this study is the first formal consideration of these dynamics.

### The models

The models developed in this report incorporate what has been learned about CRISPR-mediated adaptive immunity to phage and conjugative plasmids, primarily from the studies of Barrangou and colleagues [17] and Marraffini and Sontheimer [18], into models of the population dynamics of lytic phage [28] and conjugative plasmids [29]. Although they may appear complex, at best they are simplistic caricatures of interactions between these infectious genetic elements and bacteria with CRISPR-mediated adaptive immunity. These models are not intended or anticipated to be numerically precise analogs of these processes and dynamics.

The role of these mathematical models is similar to that of the diagrammatic models (cartoons) used to illustrate the molecular basis and mode of action of CRISPR, i.e., to provide a framework for understanding these processes, designing experiments, and interpreting their results. In this case, these experiments are on population and evolutionary dynamics of bacteria with CRISPR-mediated immunity confronted with lytic phage and competing bacteria bearing conjugative plasmids. The purpose of these models for this experimental enterprise is: (i) to identify and, in a quantitative way, evaluate the role of the different factors (parameters) contributing to these dynamics and the conditions for the establishment and maintenance of CRISPR in bacterial populations, and (ii) to generate hypotheses about these dynamics and existence conditions that can be tested (and rejected) in experimental populations.

### Predictions and some interpretations/speculations

The results of the analysis of the properties of the phage - CRISPR model are consistent with the proposition that in the presence of lytic bateriophage there are broad conditions under which a CRISPR–like adaptive immune system can become established and will be maintained in bacterial populations. With population densities, growth rates, and phage infection parameters in realistic ranges, these models predict that despite a growth rate disadvantage, bacteria with CRISPR–like acquired immunity to infecting phage will increase in frequency when initially rare and will be maintained. The necessary condition for this is that the phage population continues to persist at a sufficiently high density for CRISPR-mediated adaptive immunity to overcome an intrinsic disadvantage associated with the costs of carrying and expressing these genes.

When will the phage maintain their populations at sufficient levels for this outcome? With the parameters used to address this question, the phage will be maintained under broad conditions, but **may** eventually be lost if a population with envelope or other resistance ascends to dominance. I emphasized the word may for two reasons. The first is theoretical, if the relative growth rate of the resistant population is adequately low, the phage and thereby CRISPR will be maintained. The second is empirical, even when resistant bacteria dominate experimental populations of bacteria and phage, in general the phage continue to be maintained [30], [31], [36].

The CRISPR plasmid model predicts that because of the immunity to infection with conjugative plasmids, a lower growth rate (Malthusian fitness) CRISPR population can become established and will be maintained when competing with bacteria with a greater Malthusian fitness but bearing deleterious (fitness-reducing) conjugative plasmids. Although these conditions are met with plasmid fitness costs in the range estimated for “laboratory” plasmids [9], [37], it is not clear that naturally occurring plasmids would be as burdensome as those maintained in the Lab. The greater the Malthusian fitness burden attributed to the plasmid, the greater the advantage of CRISPR-mediated immunity.

The rate constants of plasmid transfer used in these simulations are those for plasmids with permanently derepressed conjugative pili synthesis. Wild type conjugative plasmids are more likely to be repressed for the production of these transfer organelles and would have substantially lower rates of transmission than plasmids that are permanently derepressed for plasmid transfer [38], [39]. Indeed, it is not clear whether in natural populations conjugative plasmids that engender fitness cost can be maintained by transfer alone. Their persistence may require periodic episodes where bacteria carrying them have an advantage [34], [40], but also see [41]. If the rate of infectious transfer is not sufficient to maintain deleterious plasmid in a population and they persist by continually or periodically enhancing the cells Malthusian fitness, immunity to these plasmids would not be sufficient to maintain CRISPR-encoding cells that have an intrinsic fitness disadvantage.

### Evaluating the models: estimating their parameters and testing the validity of their assumptions and predictions

It would be nearly impossible to determine whether the quantitative conditions predicted by these models for the establishment and maintenance of CRISPR-mediated immunity are met in natural populations. On the other hand, the values of the parameters of these models can be estimated and the validity of the assumptions behind their construction and hypotheses generated from the analysis of their properties can be tested in laboratory culture using CRISPR–positive and CRISPR–negative bacterial constructs, phage and plasmids of the types used respectively by Barrangou and colleagues [17] and Marraffini and Sontheimer, [18] in chemostat culture.

#### Parameters

All of the parameters of these models (Table 1 and Table 2) can be independently estimated and procedures for doing so have been published for the majority of them: (1) for the bacterial growth and resource utilization parameters, the V*_S_*, *k*, and *e*, see [26], [28]; (2) for the phage latent periods, adsorption rates δs, and burst sizes, the βs, see [28], (3) for the rate constants of plasmid transfer, the γs, see [42], [43], and (4) for the mutation rate to envelope resistance, see [44], [45]. Estimates of the plasmid segregation rate, τ, can be obtained by plating low-density cultures of plasmid-bearing cells, and testing colonies for the plasmid marker. However, unless τ is very high (τ>0.005 per cell per division), this procedure would be excessively labor intensive. However, if low, this parameter would have a negligible contribution to the dynamics of the plasmid and estimating its value would not be worthwhile.

Protocols for isolating bacteria with CRISPR-mediated resistance to phage and plasmid infection, can be found in [17] and [18], respectively. I am, however, unaware of published studies providing estimates of the fractions of phage and plasmid infected cells that become immune, the parameter *m*, or the rates of loss of these immunities, ν, in the models (Figure 2 and Figure 3). In Text S1, I outline **potential** ways to estimate these parameters. I emphasize the word potential because without actually doing these experiments, it is difficult to anticipate pitfalls and problems with the proposed procedures.

#### Assumptions and tests of their validity

In developing the model, I made a series of assumptions about CRISPR – mediated immunity and the population dynamics of bacteria with lytic phage and conjugative plasmids. In the following, I list these assumptions and briefly describe what would be anticipated experimentally if these assumptions are correct.

- CRISPR immunity to phage infection will have no effect on the rate at which phage adsorb to immune cells. If this is correct, the estimated adsorption rate parameter *δ* of a lytic phage should be the same for CRISPR cells of any immune state as well as cells of that strain for which CRISPR is non-functional.
- Phage infecting immune CRISPR cells will be lost. If this is correct, when low densities of phage are introduced into relatively high densities of exponentially growing populations of immune CRISPR cells, there should be a decline rather than an increase in the density of phage. In the model, the rate of decline in the density of phage, *P*, adsorbing to a population of bacteria with CRISPR immunity to that phage can be calculated from the estimated adsorption maximum rate parameter *δ_MAX_* and the density and maximum growth rate of bacteria, *C_R_* and *V_R_*, respectively.
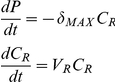
If as suggested in [17], the level of CRISPR – mediated immunity to the phage varies with the extent and nature of the phage DNA incorporated into the CRISPR region, this should be reflected as variation in the rate of loss of the phage.
- There is a multiplicity of infection (MOI) effect. When CRISPR-encoding cells are confronted with high multiplicities of phage to which they are immune, the phage will replicate and kill the immune cells. If positive results are obtained in these MOI experiments, by varying the multiplicity, the functional relationship between the MOI and the level of immunity can be determined. In doing these experiments, however, it will be necessary to rule out the possibility that those that phage that replicate on immune cells are not host range mutants [24].
- CRISPR immunity to conjugative plasmid transfer is absolute. If this is correct, the estimated rate constant of plasmid transfer γ for mixtures of donor CRISPR cells immune to that plasmid would be zero independent of the density of the culture and ratio of donors and potential CRISPR recipients. Based on the results reported in [18] as well as [17], it may well be that the level of CRISPR – mediated immunity to plasmid infection as measured by the rate constant of plasmid transfer, δ*x*, would vary with the extent and nature of plasmid DNA incorporated into the CRISPR region.
- CRISPR immunity to plasmid infection is generated during the transfer process, when the recipient first receives the plasmid, rather than during the course of plasmid carriage. If this the case, bacteria immune to plasmid transfer, C_X_, would be rare in cultures of plasmid-bearing CRISPR, the *C_P_* population. That is, they would only be generated, when C_P_ transfer the plasmid to segregants, C.

#### Population dynamics and existence conditions predictions

One way to evaluate how well these models serve as analogs of the population dynamics of bacteria with CRISPR adaptive immunity to bacteria and phage is to compare the results of simulations with independently estimated parameters to that observed in chemostat populations. Although it would be gratifying to see quantitative agreement between the anticipated dynamics and those observed in experimental populations, populations with CRISPR constructs of bacteria, conjugative plasmids and phage, it would also be surprising. These models are far too simple to expect the predicted and observed dynamics to be numerically coincident. A more modest, realistic, and, I believe, more useful goal is test predictions made from the analysis of the properties of these models in a qualitative – semi-quantitative way and identify those elements of the model that have to be modified to make the models more realistic and accurate. In the following, I list these predictions.

#### The phage model

(i) When mixtures of otherwise isogenic CRISPR positive and negative phage –sensitive constructs are introduced into chemostats in approximately equal frequencies:

- CRISPR cells with immunity to the phage will emerge and ascend to dominance.
- If the phage are maintained, the CRISPR population will continue to persist.
- If non–CRISPR mutants with envelope or other resistance to the phage evolve, or are introduced, unless they have a considerable cost in Malthusian fitness, these resistant bacteria will increase in frequency and may replace the CRISPR population.
- Although not considered in the model, there is the possibility that CRISPR cells C or C_R_ will acquire envelope resistance. If so, a CRISPR population with envelope resistance may dominate.

(ii) When introduced at low frequencies into chemostats with sensitive non–CRISPR cells in the presence of phage, as long as immune CRISPR cells are produced, the CRISPR population will increase in frequency. This will not be the case in the absence of phage.

#### The plasmid model

When mixtures of non–CRISPR cells bearing fitness reducing conjugative plasmids and plasmid-free CRISPR cells are introduced into chemostats:

- CRISPR cells with immunity to the plasmid will emerge.
- the immune CRISPR population will increase in frequency, even if the CRISPR cells have lower growth rates than plasmid-free non–CRISPR.
- the CRISPR population will decline in frequency if the environmental conditions changed so that selection favors cells bearing the plasmid. (One way to do this experiment is to use antibiotic resistance, R- plasmids and periodically add antibiotics to which the plasmid confers resistance).

### Caveats, excuses, recognized limitations, extensions, and speculations

In this report, I elected to restrict the model and its analysis to the simplest cases with lowest realistic number of states of bacteria, phage and plasmids. I have done so because at this time these minimum number of states models and the predictions generated from their analysis are more amenable to evaluating and testing experimentally than models with more states of bacteria, phage and plasmids. Moreover, these tests, and particularly the population dynamic experiments, should indicate the importance of the generation of additional population states by mutation, like host range phage and host range plasmids, are to these dynamics. Be that as it may, I also realize that this minimum number of states model will not account for what may turn out to be the most important contributions of CRISPR-mediated immunity to the ecology as well as the population and evolutionary biology of bacteria and phage.

#### Generalized resistance

Luciano Marraffini (personal communication) suggested one potentially important contribution of CRISPR to the population and evolutionary dynamics of bacteria and phage. Unlike envelope resistance, which is almost always restricted to phage that utilize single adsorption organelles, [33], CRISPR–immunity can be effective against multiple phages with different adsorption organelles (independent resistance). Moreover, envelope resistance is likely to engender a cost in Malthusian fitness, e.g. see [31], [36], [46] and that cost will almost certainly be greater if this resistance is for multiple phages that employ different receptors for infection.

If these interpretations are correct, it would seem experimental populations with CRISPR-encoding bacteria with envelope resistance to all the phage will not evolve and CRISPR will prevail in competition with sensitive non–CRISPR cells. If, however, the results of a test of this multi-phage hypothesis Ryzard Koroana and did in a study of the conditions for the maintenance of restriction endonuclease (restriction-modification, R-M) immunity are general [47], this hypothesis may be rejected. *E. coli* bearing an R-M system conferring immunity to three phage with different organelles were challenged with a mixture of all three of these phages. As a consequence of a hierarchy of phage replication [48], there was sequential selection for the different resistant states and within a day of exposure, bacteria with envelope resistance to all three phages dominated the community [47].

#### A CRISPR-mediated arms race and phage-limited communities

A number of years ago, Richard Lenski and I postulated that the arms race between bacterial resistance and host range phage would be limited to few cycles and is likely to end with resistant bacteria to which phage would not be able to generate host range mutations [46]. The empirical basis of our hypothesis was the results of experiments with *E. coli* and its phage and envelope resistance, [31], [46], [49], [50]. While this interpretation was also supported by experiments with *V. cholerae* and its phage JSF4 [36], experiments with *Pseudomonas fluorescens* and its phage SBW25 [51] suggest extended arms races are possible. Although, to my knowledge, the mechanisms responsible for the continuous changes in resistance and host-range reported in this study with this strain of Pseudomonas and phage have yet to be elucidated, CRISPR does provide a mechanism for long-term arms races between bacteria and phage [21], [22], [24]. By single base changes in sequences of DNA into the spacer regions of CRISPR, a phage can infect and replicate on previously immune CRISPR cells. By incorporating the mutated or other region of that phage into another spacer, CRISPR cells can generate resistance to these host range phages. At this time, it is not at all clear how long or through how many cycles a CRISPR-mediated arms race can proceed. I would it certainly be interesting, tenable experimentally and fun to find out. Be it by CRISPR or by sequential resistance and host-range mutation [52], [53] an extended arms race could provide a way for phage, rather than resources, to limit the densities of bacterial populations (see Text S2), which is an ecological outcome with practical as well as theoretical implications, e.g. see [54]–[59].

## Supporting Information

Text S1 Protocols to estimate the probability of formation, *m*, and rate of loss, ν, of CRISPR-mediated immunity to phage and conjugative plasmids.
(0.03 MB DOC)

Text S2 Arms races and phage-limited bacterial populations.
(0.33 MB DOC)
