# Supplementary material for: Nasty Viruses, Costly Plasmids, Population Dynamics, and the Conditions for Establishing and Maintaining CRISPR-Mediated Adaptive Immunity in Bacteria
Source: PLoS Genet. 2010 Oct 28;6(10):e1001171. doi: 10.1371/journal.pgen.1001171 (PMC2965746; doi:10.1371/journal.pgen.1001171)
Supplement: Text S1 — Protocols to estimate the probability of formation, m, and rate of loss, ν, of CRISPR-mediated immunity to phage and conjugative plasmids. (0.03 MB DOC) [file pgen.1001171.s001.doc]

**Supporting Information 1: Protocols to estimate the probability of formation, *m,* and rate of loss, ν, of CRISPR mediated immunity to phage and conjugative plasmids.**

1- To estimate the fraction of phage-infected CRISPR that become immune, *m,* the latent period and adsorption rate, δ, of the phage on non-immune CRISPR cells should first be estimated. Given δ and the densities of phage and bacteria exposed, P and C, the number of bacteria infected over a period less than the latent period can be calculated from the model. The fraction of phage-exposed C that acquired immunity C_R_ can then be estimated by plating on agar with high densities of phage. For this estimator we’d have to assume envelope resistance is rare relative to immunity and there is little or no MOI effect killing the immune cells on the plate.

2- Obtaining an accurate independent (rather than fitted) estimate of *m* for the plasmid model would be more difficult than that for the phage model and may not be possible unless *m* is relatively high (say *m> 0.01)* and the plasmids used transfer at high rates. In the model I assumed that CRISPR-mediated immunity to plasmids occurs during transfer, rather than after the plasmid becomes established. If this is the case, the fraction of cells that acquire the plasmid and become immune to it, the parameter *m,* can be calculated from the densities of plasmid donors and CRISPR recipients, *N_P_* and C in the model, and estimates of the rate constant of plasmid transfer, δ_NC_ and exponential growth rates, *V_NP_* and *V_C_*. To estimate m, genetically marked donors and recipients, N_P_ and C would be grown together for a defined period of time and their densities and those of the transconjugants, *C_P_* estimated by selective plating. With a high transfer rate plasmid and high enough densities, the majority of *C* recipients would acquire the plasmid, be converted into C_P_. The anticipated density of transconjugants can be calculated from a model of the dynamics of plasmid transfer [38]. A fraction of the C in these cultures would be immune to the plasmid, C_X_ in the model. To estimate that fraction and calculate m, it will be necessary to estimate the proportion of recovered non-transconjugant CRISPR cells, C+C_X_, that are refractory to the plasmid, C_X_. This could be done by testing for the transfer of the plasmid by adding N_P_ cells to growing microtitre cultures established from C+C_X_ colonies obtained from these experiments. Those refractory to the plasmid transfer are C_X_.

3- To estimate the rate of loss of CRISPR-mediated phage resistance, *v*, a growing population of C_R_ would be sampled and colonies screened for resistance to the phage. For some phages, this screening can be done by “toothpicking” colonies across streaks of phage. For others, it may be necessary to spot test on small soft agar lawns or to test for the replication of the phage in microtitre cultures to which low densities of phage are added.

4- To estimate the rate of loss of immunity to the plasmids, ν, Cx 🡪 C, using the microtire plate procedure described above to test for the inability to receive the plasmid, colonies of a growing culture of *Cx* would be tested for the ability to receive the plasmid.
