# Supplementary material for: Nasty Viruses, Costly Plasmids, Population Dynamics, and the Conditions for Establishing and Maintaining CRISPR-Mediated Adaptive Immunity in Bacteria
Source: PLoS Genet. 2010 Oct 28;6(10):e1001171. doi: 10.1371/journal.pgen.1001171 (PMC2965746; doi:10.1371/journal.pgen.1001171)
Supplement: Text S2 — Arms races and phage-limited bacterial populations. (0.33 MB DOC) [file pgen.1001171.s002.doc]

**Text 2. -Arms Races and Phage-Limited Bacterial Populations**

In this appendix I illustrate how sequential changes in the immunity (resistance) states and host range mutation in phage (or multiple phage) can maintain an extended arms race and phage-, rather than resource- limited bacterial populations. For this I use a chemostat phage-bacteria population dynamic model similar to that employed for the CRISPR model, but assume resistance is generated by mutation rather than acquired through phage infection In this model there is a single resource of concentration R and n states of bacteria, Ni, and phage, Pj,(i, j = 1,2,3, …n). Phage of state j adsorb to bacteria of state i with a rate constant ij and produced βij­ phage j particles with each infection. The bacteria grow at maximum rates, Vi, and all require *e* units of the resource (conversion efficiency) to produce a cells and have the same Monod constant, k, and are washed out at the same rate w. Neglecting the latent period and an MOI effect, the rates of change in the densities of bacteria and phage and concentration are given by,

Numerical solutions to these equations (computer simulations) were programmed in Berkeley MadonnaTM and are available on [www.eclf.net/programs](http://www.eclf.net/programs). In these simulations I allow for a maximum of 5 states, n=5, and assume a refuge density below which the phage cannot replicate and the bacteria survive and mutations to different bacterial and phage states occur at random via a Monte Carlo process with all states equally likely to be produced. For a similar model and a more comprehensive formal (mathematical) considerations of this co-evolutionary process see [1] and [2].


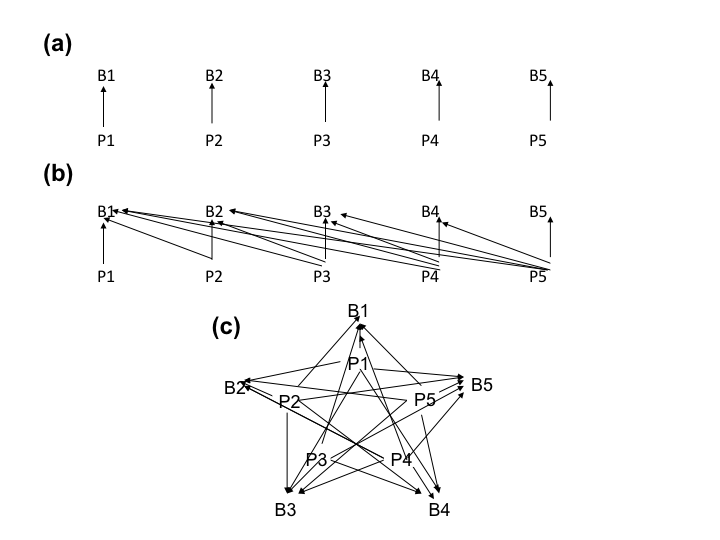


**Figure S1.** Three scenarios for an envelope resistance and host-range arms race for five states of bacteria and phage. (a) Unique receptors; each bacterial state is infected by a single phage state and each phage state is capable of replicating on only a single state of bacteria. (b) Host range phage are capable of replication on bacteria with the same index number and earlier states (lower index numbers). (c) No universal resistance - phage are capable of replicating on bacteria of all states.

The three scenarios of immunity (resistance) – host range relationships considered in the simulation runs presented below are illustrated in Figure S1.


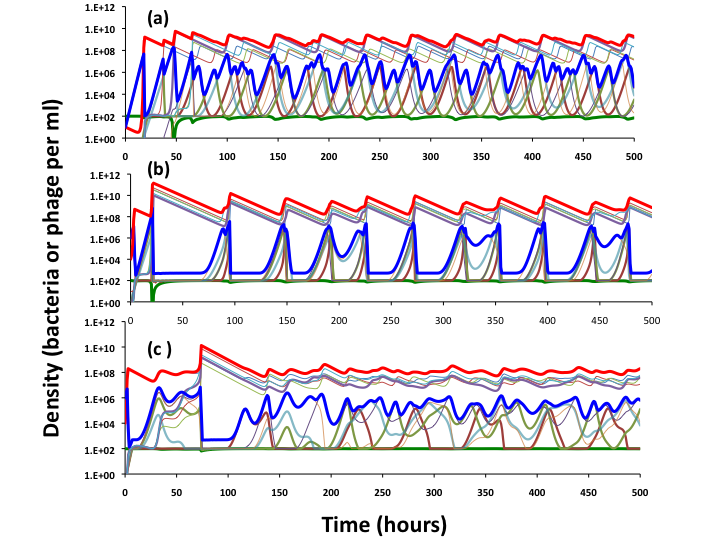


**Figure S2.** Population dynamics of multiple phage and bacterial states, Arms Race Model; changes in the densities of bacteria and phage and the concentration of the limiting resource. Thick red line- total phage density; thick blue line -density of bacteria; thick green line - concentration of the limiting resource. The lighter lines are the densities of the different phage and bacterial populations. (a) Unique receptors, each bacterial state is infected by a single phage and each phage is capable of replicating on only a single state of bacteria. (b) Host range phage are capable of replication on bacteria with the same index number and earlier states (lower index numbers). (c) No universal resistance, phage are capable of replicating on bacteria of all states (see Figure A1 for the diagrams of these scenarios). At time 0 in all these simulations bacteria and phage of only the first state were present, B1 and P1. The parameter values employed for these simulations are listed in Table S1.

As can be seen in Figure S2, as long as the resistant mutants are countered by host range phage, under each of these three scenarios, the community at large remains phage- rather than resource limited. That is the average density of bacteria remains well below that anticipated for the phage-free chemostat.

**Table S1. Parameter Values for Figure S2**

Common parameters

e=5x10^-7^, k=4.0, w=0.10, A=100, mutation rate to a new bacterial state µB=10-7, mutation rate to a new host range state, µP=10-7.

V1=1.00, V2=0.95, V3 =1.00, V4=0.80, V5 =1.0

All the burst sizes, βij =30, i, j=1,2,3,4,5

Adsorption rates

(a) Unique receptors

Phage

| Bacteria | 1 | 2 | 3 | 4 | 5 |
| --- | --- | --- | --- | --- | --- |
| 1 | 1.0E-08 | 0.0E+00 | 0.0E+00 | 0.0E+00 | 0.0E+00 |
| 2 | 0.0E+00 | 2.0E-08 | 0.0E+00 | 0.0E+00 | 0.0E+00 |
| 3 | 0.0E+00 | 0.0E+00 | 1.5E-08 | 0.0E+00 | 0.0E+00 |
| 4 | 0.0E+00 | 0.0E+00 | 0.0E+00 | 1.0E-09 | 0.0E+00 |
| 5 | 0.0E+00 | 0.0E+00 | 0.0E+00 | 0.0E+00 | 5.0E-09 |

(b) Replication on bacteria with the same or lower index numbers

| Bacteria | 1 | 2 | 3 | 4 | 5 |
| --- | --- | --- | --- | --- | --- |
| 1 | 1.0E-09 | 2.0E-09 | 3.0E-09 | 4.0E-09 | 1.0E-09 |
| 2 | 0.0E+00 | 2.0E-09 | 3.0E-09 | 5.0E-09 | 6.0E-09 |
| 3 | 0.0E+00 | 0.0E+00 | 7.0E-09 | 1.0E-09 | 2.0E-09 |
| 4 | 0.0E+00 | 0.0E+00 | 0.0E+00 | 1.0E-09 | 3.0E-09 |
| 5 | 0.0E+00 | 0.0E+00 | 0.0E+00 | 0.0E+00 | 2.0E-09 |

(c) No universal resistance

| Bacteria | 1 | 2 | 3 | 4 | 5 |
| --- | --- | --- | --- | --- | --- |
| 1 | 5.0E-08 | 4.0E-08 | 3.0E-08 | 2.0E-08 | 1.0E-08 |
| 2 | 5.0E-08 | 4.0E-08 | 3.0E-08 | 2.0E-08 | 1.0E-08 |
| 3 | 5.0E-08 | 4.0E-08 | 3.0E-08 | 2.0E-08 | 1.0E-08 |
| 4 | 5.0E-08 | 4.0E-08 | 3.0E-08 | 2.0E-08 | 1.0E-08 |
| 5 | 5.0E-08 | 4.0E-08 | 3.0E-08 | 2.0E-08 | 1.0E-08 |

equilibrium and the concentration of resource remains at nearly the level of the reservoir. Although the bacteria differ in their relative fitness (maximum growth rates) and the phage have different adsorption rate constants, the diversity of the bacterial and phage populations are maintained, although the densities of the different states continue to vary.

**REFERENCES**

1. Weitz JS, Hartman H, Levin SA (2005) Coevolutionary arms races between bacteria and bacteriophage. Proc Natl Acad Sci U S A 102: 9535-9540.

2. Forde SE, Beardmore RE, Gudelj I, Arkin SS, Thompson JN, et al. (2008) Understanding the limits to generalizability of experimental evolutionary models. Nature 455: 220-223.
